# Reviews and Book Notices

**Published:** 1883-06

**Authors:** 


### Reviews and Book Notices.

---

ARTICLE IX.—A TREATISE ON THERAPEUTICS, comprising Materia Medica and Toxicology, with especial reference to the Application of the Physiological Action of Drugs to Clinical Medicine. By H. C. WOOD, Jr., M.D., Prof. of Materia Medica and Therapeutics in the University of Pennsylvania, etc., etc. Fourth edition. Revised and enlarged. 8vo, pp. 736. Philadelphia: J. B. Lippincott & Co. 1882. Chicago: Jansen, McClurg & Co.

This is one of the leading text books on the subject. It is divided in two very unequal parts, viz.: Drugs, and Forces, by which is meant heat and cold, and electricity. The drugs are divided into systemic and non-systemic remedies, the latter being understood of antacids, disinfectants, etc. Systemic remedies comprise general remedies, like the tonics and antispasmodics, alteratives, etc., and local remedies like cathartics, diuretics, emollients, etc. The propriety of such divisions is not very obvious; for instance, to call a poultice a systemic remedy and call an antacid like lime a non-systemic agent, does not seem rational, at least it is not clear.

The author undoubtedly possesses a great deal of discriminative judgment, but presents together the most opposite views of the various experimenters, reserving his decision when their experiments seem not to legitimate their views. The large proportion of the book given to such contradictory statements make the general ignorance of the therapeutic action of drugs quite obvious. One would wish to read more of the author's experiments and of his conclusions, for on such debatable subject innovations are of more value than mere conservative views.

In this, perhaps, the work under consideration is susceptible of much improvement. There is, for instance, the unpardonable omission of the metric system in stating the doses of the various drugs. Very little is said as to the mode of administration of unpalatable medicines, like opium, quinine, etc., that is, so-called elegant preparations, are never alluded to. Many of the latest applications of certain old drugs have been overlooked; for instance, the use of quinine as a stimulant, by so many people, in lieu of alcohol, etc. Under the head of Forces, one vainly expects to find Massage and the Swedish movement treated of. But the strangest incensistency of the author is his partial disbelief in the dependence of all infectious diseases on organic germs. But as there are so few reliable works on Therapeutics, and that valuable original contributions like Bartholow's Antagonism, appear once in a decade at best, we take pleasure in recommending the use of the present volume to students, and its correction and improvement to the author. H. D. V.

---

ARTICLE X.—MANUAL OF GYNÆCOLOGY. Hart and Barbour. Two Vols. (Wood's Library, January, 1883.)

In the preface of this work, the authors avow their belief in anatomy, physiology and pathology of the tissues and organs under discussion, as being the foundation of a clinical text-book. The reader of these volumes will hardly fail to remark the well-ordered manner in which the authors' belief is exemplified. Anatomical descriptions are based upon the results obtained by the authors and different observers employing frozen sections; structural anatomy and physics of the pelvis are clearly stated; while menstruation and ovulation receive a concise and practical description. Plain and practical directions regarding instruments and methods employed in examinations follow. The slippery-elm test is not mentioned, although a favorite with some American writers.

The diseases of the pelvic tissues, beginning with the peritoneum and connective tissue, are treated of in a systematic and thorough manner; the details of treatment are fully described; the whole result being the presentation of conclusions and results, with practical points, in a manner easy of access and remarkably clear. The pathology of the lining membrane of the uterus is accurately stated. The common affection known as "ulceration" is described as "chronic cervical catarrh," and the name explained by the microscopic study of recent observers.

Ruptures of the perineum are explained and treated with reference to the physics of the pelvic floor; in uterine displacements from defective support, prolapsus is considered a hernia analogous to femoral hernia.

The operations for vesico-vaginal fistula are fully described and illustrated. An appendix treats of specific disease; chlorosis; etiology of uterine disease; the recording of cases, and a *résumé* of gynæcological literature completes the work.

Quotations from German, English and French literature abound. A brief bibliography heads every chapter.

The volumes are copiously illustrated. While some cuts are obscure, the greater portion are plain, and there are several excellent lithographs. For conciseness, clearness and practical information, the work commends itself to the student and practitioner, and forms a very desirable portion of Wood's excellent library. E. P. D.

---

ARTICLE XI.—A TREATISE ON ALBUMINURIA. BY W. H. DICKINSON, M. D. Second edition, 8vo. cloth, pp. 300. New York: William Wood & Co. 1881.

This is the January, 1881, issue of Wood's Library of Standard Medical Authors. It is remarkable for its six beautifully colored lithographic plates, representing various diseased conditions of the kidneys, together with thirty-one wood cuts, in the text. The subject matter comprises chapters on nephritis or Bright's Disease, granular degeneration, lardaceous degeneration, and considerations pertaining thereto.

The pathology of diseases of the kidney is considered at length, and micrographs illustrating the same render the study of the subject so much easier. As to treatment, the author gives a synopsis of that advocated by his predecessors, then he warns one against bleeding, recommends drinking water to dilute the urine, and restricts the use of nitrogenous food to a minimum. He does not favor the indiscriminate use of digitalis. Purgatives form part of the preventive treatment of nephritis. Iron is recommended and gallic acid discarded. Paracentesis seldom required. Baths are of much benefit.

This is one of the valuable volumes of the series in which it belongs, and will repay the small investment with interest.

H. D. V.

---

ARTICLE XII.—LECTURES ON DISEASES OF CHILDREN; a Handbook for Physicians and Students. BY DR. EDWARD HENOCH, Prof. in the University of Berlin. 8vo, cloth, pp. 357. New York: William Wood & Co. 1882. The March issue of Wood's Library.

The value of this manual rests on the extensive experience of the author, which is supported by the timely insertion of reports of cases, and contains much valuable literature found nowhere else. Notwithstanding the contrary anticipation, one finds a number of diseases often met in general practice treated here at length, which are not to be met in more classical works on the same subject. Yet, some diseases are left out purposely, as variola, nor are affections of the eye and ear mentioned, but there are a few pages given to skin diseases.

The treatment recommended is that peculiar to the German school, so well represented in Ziemssen's Cyclopœdia; it consists of few but effective measures, and is well adapted for the busy practitioner. Although, perhaps, more valuable in a few respects, it is not at all likely that this book will replace the well known American treatises on the diseases of childhood, partly on account of its being adapted to a so differently constituted people, but at the same time it will remain one of the most desirable books in Wood's Library. H. D.V.

ARTICLE XIII.—CLINICAL LECTURES ON THE DISEASES OF OLD AGE. BY J. M. CHARCOT, M D., and ALFRED L. LOOMIS, M.D. 8vo. cloth, pp. 280. New York: William Wood & Co. 1881. June No. of Wood's Library.

Gout and chronic articular rheumatism are the diseases treated of by Prof. Charcot, while Dr. Loomis' lectures treat of pneumonia, catarrh, asthma, atheroma, apoplexy, constipation, etc. The introduction gives the history of the subject in an able manner, and is quite interesting, and so is the lecture on the general characteristics of senile pathology. Yet, the prolixity peculiar to French writers is manifest here, and makes the reading of some chapters rather tedious, although eminently erudite. As medical works of this character are quite rare, the publishers deserve the thanks of the profession for issuing this one in such a cheap and accessible form. H. D. V.

---

DR. ANDERS reported to the society of physicians at St. Petersburg his results on the treatment of spondylitis with the felt-jacket. He takes first a model of the upper body with plaster Paris, and he has the felt jacket made exactly after this model. He claims superiority for the felt jacket over the plaster Paris jacket of Sayre; and he says that, especially to children, the latter apparatus is not applicable. He has treated fifty patients affected with spondylitis in all stages of the disease. He had some who had abandoned walking for several months. and he says that his felt jackets have been always easily worn and that every case was followed by the most decided success.—*St. Petersburg Wochenchr.*

To remove fish-bones from the throat Prof. Voltolini, at Breslan, recommends a gargle composed of muriatic acid, 4 parts; nitric acid, 1 part; and water, 240 parts. The teeth have to be protected by lard or oil. The fish-bones become flexible and they disappear entirely after a short time.—*Memorabilien.*

